# Analgesic efficacy of parasternal intercostal plane block for midline sternotomy in adult cardiac surgery: A systematic review and meta-analysis of randomized controlled trials

**DOI:** 10.14440/jbm.2024.0070

**Published:** 2024-11-14

**Authors:** Heitor J. S. Medeiros, Amanda Cyntia Lima Fonseca Rodrigue, Ariel Mueller, Elizabeth Korn, A. Sassan Sabouri

**Affiliations:** 1 Department of Anesthesia, Critical Care and Pain Medicine, Massachusetts General Hospital Boston, Boston, Massachusetts, 02114, United States of America; 2 Department of Medicine, Positivo University, 81280-330 Curitiba, Paraná, Brazil

**Keywords:** Regional anesthesia, Acute pain, Cardiac surgery, Median sternotomy, Pain management, Ultrasonography

## Abstract

**Background:**

Regional anesthesia is widely supported as a part of multimodal analgesia for post-operative pain management following cardiac surgery. A common technique for managing post-sternotomy pain is the parasternal intercostal plane (PIP) block, which involves injecting anesthetics into the fascial planes near the sternum to block the anterior cutaneous branches of the T2 – T6 nerves.

**Objective:**

This study aimed to assess the effects of PIP blocks on post-sternotomy pain, narcotic usage, intensive care unit (ICU) stay, and extubation time following adult cardiac surgeries.

**Methodology:**

We conducted a systematic review and meta-analysis of randomized controlled trials (RCTs) investigating PIP blocks in adult cardiac surgery with midline sternotomy. Studies were retrieved from PubMed, Cochrane Library, and Embase through February 22, 2023. The risk of bias (ROB) in RCTs was assessed using the Cochrane ROB tool, version 2. Twelve RCTs involving 819 adult patients were included. Primary outcomes were pain scores at 12 and 24 h post-surgery and narcotic usage in morphine milligram equivalents (MME). Secondary outcomes included extubation time and ICU stay.

**Results:**

The PIP block group had significantly lower pain scores at 12 (mean difference [MD]: −1.21 points, 95% confidence interval [CI]: −2.17, −0.25, *p* = 0.013) and 24 hours (MD: −0.69 points, 95% CI: −1.35, −0.02, *p* = 0.042), and reduced MME use (MD: −30.34 MME, 95% CI: −45.80, −14.89, *p* < 0.001). PIP blocks did not significantly reduce extubation time (MD: −0.77 h, 95% CI: −1.64, 0.09, *p* = 0.080) but were associated with shorter ICU stay (MD: −0.54 days, 95% CI: −0.94, −0.13, *p* = 0.009).

**Conclusion:**

PIP blocks provided effective analgesia and reduced ICU stay in cardiac surgery patients requiring sternotomy, but due to study heterogeneity, results should be interpreted with caution. Future research is warranted to explore its short- and long-term outcomes.

## 1. Introduction

Post-operative pain following cardiac surgery is a significant source of short- and long-term patient distress, increased cost of patient care, chronic pain, and disability.^1^ Acute pain after cardiac surgery arises from a combination of somatic, nociceptive, inflammatory, visceral, and neuropathic factors, often exacerbated by the body’s inflammatory stress response.^2^ The primary contributors to this acute pain include the surgical incision, sternotomy, thoracotomy, and tissue retraction, which may lead to rib fractures or dislocation of the costochondral and costovertebral joints. In addition, nerve damage resulting from retraction, dissection, or surgical positioning plays a critical role.^3^ Typically, the most intense pain occurs on the first post-operative day, gradually diminishing by the 3^rd^ day.^4^

Evidence suggests that different regional anesthesia techniques offer numerous benefits when integrated into a multimodal analgesic strategy for cardiac surgery, including superior pain relief, decreased reliance on opioids or sedatives, expedited extubation with improved respiratory function, better hemodynamic stability, and reduced stress response during the perioperative phase.^5^ Regional anesthesia encompasses a wide array of techniques, including fascial plane blocks, which have increasingly been used to manage acute post-cardiac surgery pain.^4^,^5^ One of the most commonly employed techniques for post-sternotomy pain is the parasternal intercostal plane (PIP) block, which has been shown to provide effective analgesia and lower opioid consumption after surgery.^6^-^8^ The PIP blocks may be performed either deeply or superficially, targeting different anatomical planes. The superficial PIP block involves the space between the internal intercostal muscles and the ribs, lying beneath the pectoralis major muscle and lateral to the sternum.

This technique is known by various names, including PIP block, pectointercostal fascial plane block, subpectoral interfacial plane block, and parasternal pecs block.^9^ In contrast, the deep PIP block targets the area between the internal intercostal muscle and the transversus thoracis muscle, and is also referred to as the transversus thoracis muscle plane block. Both techniques aim to anesthetize the anterior cutaneous branches of the intercostal nerves, as highlighted by recent cadaveric studies^10^,^11^ evaluating the dispersion of the anesthetic under the nerve’s cutaneous branches.

This systemic review and meta-analysis evaluated the analgesic impact of PIP blocks following open cardiac surgery involving mid-sternotomy in adult patients under general anesthesia. The research scope was defined against the population, intervention, comparison, outcomes, and study design^12^ framework, which includes the following components: The population consisted of adult patients over 18 years old who underwent cardiac surgery with midline sternotomy; the intervention was the PIP blocks for post-sternotomy analgesia; the comparison involved patients that did not receive a PIP block or received a placebo PIP block; the primary outcomes included pain levels in the first 24 h post-surgery and narcotic usage measured in morphine milligrams equivalent (MME), and the secondary outcomes comprised time to extubation and length of stay in the intensive care unit (ICU). This systematic review especially focused on randomized controlled trials (RCTs) investigating the analgesic effects of PIP blocks.

## 2. Methods

This study was designed as a meta-analysis of RCTs, adhering to the 27-step guide and the Preferred Reporting Items for Systematic Reviews and Meta-Analysis (PRISMA) guidelines for conducting and reporting systematic reviews.^13^ The protocol was registered with the PROSPERO database (CRD42023403228).

### 2.1. Search strategy and information sources

**Figure 1:**
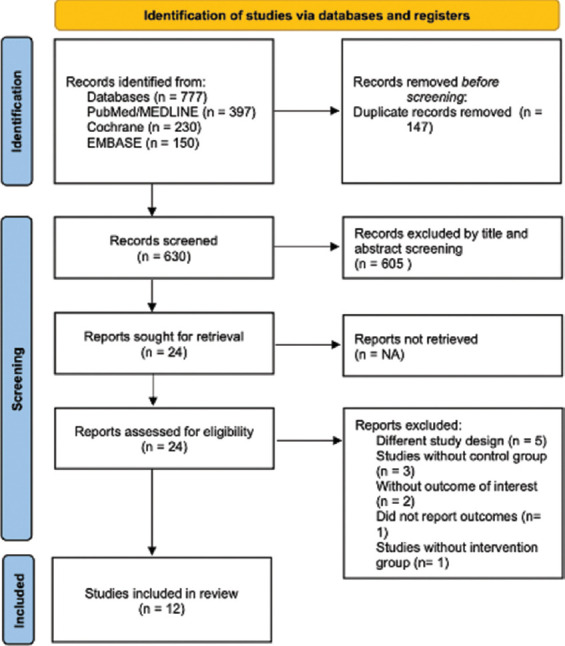
Flow diagram of study screening. This flowchart illustrates the process of study, detailing the included studies, the databases searched, and the reasons for exclusion at each stage.

We performed a literature search in PubMed, Cochrane Library, and Embase databases from inception through February 22, 2023, to identify studies published in English. Two reviewers (HJSM and ACLFR) screened the titles and abstracts to identify potentially relevant articles. Discrepancies in judgment were resolved through discussion. Abstracts of complete studies and conferences, as well as clinical trial registries, were evaluated for relevance and the presence of outcomes of interest. Based on this evaluation, a list of compatible studies was compiled. The included studies were then evaluated by a third reviewer (AS). A flow diagram of the screening process is illustrated in Figure 1. We also documented any reported complications. The search strategy utilized terms such as “Parasternal intercostal plane block,” “Cardiac surgery,” and “Randomized controlled trial,” with Boolean operators used to combine search terms. The complete strategy is detailed in the Supplementary File. Data extraction was conducted independently by two reviewers (HJSM and ACLFR) using standardized forms, with discrepancies resolved through discussion.

#### 2.1.1. Eligibility criteria

RCTs were included if they compared the intervention (PIP block) with either a placebo or no regional anesthesia. Eligible studies involved cardiac surgeries with midline sternotomy, and results had to be presented in English. The superficial PIP block, previously referred to as PIP block, also known as the pectointercostal fascial plane block, subpectoral interfascial plane block, or parasternal pecs block, targets the plane superficial to the internal intercostal muscles and ribs, and deep to the pectoralis major muscle lateral to the sternum. The deep PIP block, or transversus thoracic muscle plane block, targets the space between the internal intercostal muscle and transversus thoracic muscles.^9^ Both single shots and continuous block techniques, whether ultrasound-guided or landmark-based, were evaluated.

#### 2.1.2. Exclusion criteria

We excluded studies based on the following criteria: (i) studies involving patients under 18, (ii) studies that did not employ PIP blocks, (iii) studies with overlapping populations, defined as samples from the same institutions and recruitment periods, (iv) studies that did not report at least one primary and secondary outcomes, including pain scores at 12 and 24 h post-surgery, MME usage, time to extubation, and length of stay in the ICU, and (v) non-randomized studies, as we focused exclusively on RCTs. No restriction was placed on publication year, and authors were contacted for missing data.

### 2.2. Definition of outcomes

The primary outcomes included pain scores at 12 and 24 h post-surgery, assessed on the visual analog scale and numeric rating scale, both utilizing a 10-point scoring system. In addition, all data on opioid consumption during the first 24 h were standardized to oral MME using a conversion calculator.^14^ This standardization allows for more accurate comparisons across studies, given the varying potencies of different opioids that cannot be directly compared without this adjustment. Secondary outcomes comprised the length of stay in the ICU measured in days and the time to extubation recorded in hours. The effect measures for each outcome included mean difference (MD) for pain scores and MME consumption, as well as risk ratio for the time to extubation and ICU length of stay.

### 2.3. Risk of bias (ROB) in individual studies

The ROB in RCTs was assessed using the Cochrane ROB tool, version 2 (ROB 2),^15^ which is recommended by the Cochrane Collaboration. Two independent reviewers (HJSM and ACLFR) evaluated each domain for ROB, with discrepancies resolved by comparing notes. Publication bias was rated by creating funnel plots of the treatment effects estimated from the included studies.

Each RCT was evaluated based on the following parameters: (1) concealment of the allocation sequence, (2) blinding of participants, healthcare providers, data collectors, and outcome adjudicators, (3) termination of the trial, if applicable, (4) the percentage of patients lost to follow-up, and (5) adherence to the intention-to-treat principle.

### 2.4. Statistical analysis

We compared treatment effects for continuous outcomes using MD and for categorical outcomes using relative risk with a 95% confidence interval (CI). All data analyses were performed using RStudio, version 4.2.3.,^16^ utilizing the base package, as well as the “meta” and “metafor” packages.^17^ A random-effects model with restricted maximum likelihood estimation was employed to account for clinical heterogeneity among the included studies. Pooled MDs were reported as summary measures, and forest plots were generated to display the results. A meta-regression analysis was conducted for each primary outcome, evaluating the effects of publication year, procedure type, and timing of block placement (before or after surgery). Given the limited number of studies, this analysis was reported using descriptive statistics only. To assess the influence of individual studies on the overall effect size, a leave-one-out sensitivity analysis was performed, removing one study at a time and repeating the analysis. A significance threshold of *p* < 0.05 was applied, with non-overlapping 95% CI considered statistically significant.

## 3. Results

### 3.1. Search results

Following the search strategy outlined in Figure 1, a total of 24 unique articles were assessed for eligibility. Ultimately, 12 RCTs involving 819 patients were included in the analysis, with six studies specifically examining the primary outcomes of interest.

Study characteristics and overviews are summarized in Table 1. The authors opted to present the regional techniques using the current nomenclature established by the American Society of Regional Anesthesia and the European Society of Regional Anesthesia consensus,^9^ rather than the terminology used by the original authors. Notably, across all studies, no complications related to regional anesthesia were reported.

**Table 1:** Overview of included studies

Study	Study type and sample	Population	Intervention	Control	Post-operative Analgesia	Outcome of interest
Aydin *et al*., 2020^18^	RCT; double-blind; *n*=48; intervention=24; control=24	Cardiac surgery	Deep PIP, single shot (20 mL Bupivacaine 0.25%) after induction.	Deep PIP, single shot (20 mL of 0.9% saline solution) after induction.	Tramadol 1 mg/kg iv if VAS≥4	(1,2,3,4)
Baki *et al*., 2016^19^	RCT; double blind; *n*=81; intervention=45; control=43	CABG surgery	Superficial PIP single shot, before sternal wire placement (50 mL Levobupivacaine 0.25% and fentanyl 100 mcg)	No block	Tramadol PCA iv – 10 mg/h continuous dose+20 mg as needed+tenoxicam IV	(3)
Barr *et al*., 2007^20^	RCT; double-blind; *n*=81; intervention=45; control=43	Non-emergency primary multivessel CABG surgery on or off cardiopulmonary bypass, AVR, MVR, atrial septal defect repair.	Superficial PIP single shot (40 mL Ropivacaine 0.75%) before sternal closure	Superficial PIP single shot (40 mL of 0.9% saline solution)	Morphine PCA iv as needed+NSAIDs iv+tramadol 50 – 100 mg iv as needed	(4)
Bloc *et al*., 2021^21^	RCT; double-blind; *n*=35; intervention=18; control=17	CABG surgery	Superficial PIP single shot (60 mL Ropivacaine 0.25%) after induction	Superficial PIP single shot (60 mL of 0.9% saline solution)	Not available	(4)
Hamed *et al*., 2022^22^	RCT; triple blind; *n*=70; intervention=35; control=35	CABG or valve replacement surgery	Superficial PIP single shot (20 mL Bupivacaine 0.25%) at the end of the surgery	Superficial PIP single shot (dry needling)	Morphine iv 5 – 10 mg as needed	(1,2,3,4,5)
Hamed *et al*., 2022^23^	RCT; double-blind; *n*=70; intervention=35; control=35	Valve replacement or adult congenital surgery	Deep PIP, single shot (20 mL Bupivacaine 0.25%) at the end of the surgery	Deep PIP, single shot (20 mL of 0.9% saline solution)	Fentanyl PCA iv (max 90 mcg/h, no push dose)	(1,2,3,4)
Khera *et al*., 2021^24^	RCT; quadruple; blind; *n*=80; intervention=40; control=40	CABG and/or valve replacement surgery	Superficial PIP, two shots (20 mL Bupivacaine 0.25%) within 2 h after admission on ICU	Superficial PIP, two shots (20 mL of 0.9% saline solution)	Lidocaine patches	(5)
Krishnan *et al*., 2022^25^	RCT; double blind; *n*=60; intervention=20; control=27	CABG	Superficial PIP single shot (40 mL Bupivacaine 0.25%) before chest closing in the OR	Superficial PIP single shot (4 0mL of 0.9% saline solution)	Not available	(1,2 3,4,5)
Kumar *et al*., 2021^26^	RCT; single blind; *n*=40; intervention=20; control=20	Cardiac surgery	Superficial PIP single shot (20 mL ropivacaine 0.25%) before discharge from ICU	No block	Acetaminophen 1 g+tramadol 50 mg iv every 6 h	(1,4)
Shokri *et al*., 2021^27^	RCT; double blind; *n*=60; intervention=30; control=30	Cardiac surgery	Deep PIP, single shot (30 mL of 0.25% bupivacaine) after induction	Deep PIP, single shot (30 mL of 0.9% saline solution) after induction	Acetaminophen 1 g iv every 6 h	(1,2,3,4,5)
Vilvanathan *et al*., 2020^6^	RCT; double blind; *n*=90; intervention=45; control=45	CABG	Superficial PIP, single shot (20 mL levobupivacaine 0.5%) after induction	No block	Tramadol 100 mg iv if NRS>3	(4)
Zhang *et al*., 2022^7^	RCT; double blind; *n*=116; intervention=55; control=55	Cardiac surgery	Superficial PIP, continuous (40 mL ropivacaine 0.33%) before induction	Superficial PIP, continuous (40 mL of 0.9% saline solution) before induction	Sufentanil 0.05 mcg/kg iv every 4 h as needed+flurbiprofen 100 mg iv as needed	(2,3)

Notes: The outcomes of interest are listed and abbreviated in the respective column in the following order: (1) post-operative pain scores at 12 h, (2) post-operative pain scores at 24 h, (3) MME consumption, (4) time to extubation, and (5) intensive care unit length of stay.

Abbreviations: AVR: Aortic valve repair; CABG: Coronary artery bypass surgery; iv: Intravenous; mcg: Microgram; MVR: Mitral valve replacement; N: Total sample; NRS: Numeric rating scale; NSAIDs: Non-steroidal anti-inflammatory drugs; OR: Operating room; PCA: Patient-controlled analgesia; PIP: Parasternal intercostal plane block; RCT: Randomized controlled trial; SD: Standard deviation; VAS: Visual analog scale.

### 3.2. ROB and meta-regression

**Figure 2:**
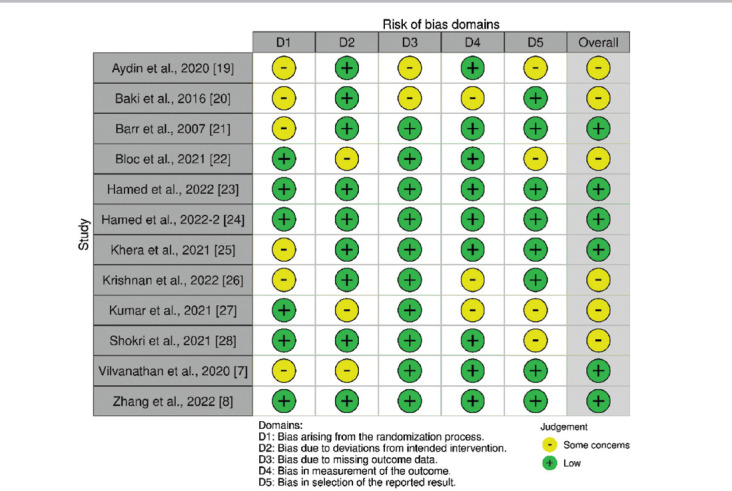
Assessment of the risk of bias for the included studies, evaluated using version 2 of the Cochrane risk of bias tool. Notes: D1: Bias arising from the randomization process; D2: Bias due to deviations from intended intervention; D3: Bias due to missing outcome data; D4: Bias in measurement of the outcome; D5: Bias in the selection of reported results.

A summary of the ROB for each domain is presented in Figure 2. Most studies exhibited low risk or minor concerns regarding bias. Allocation concealment and blinding of participants and personnel were consistently rated as low risk.

**Figure S1:**
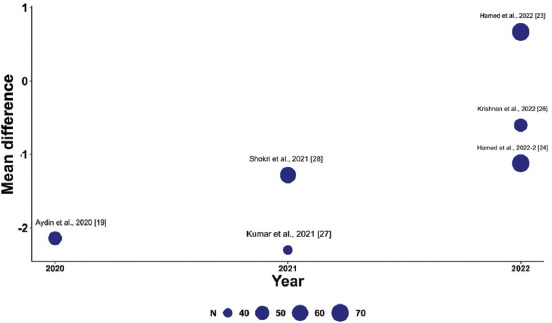
Meta-regression showing the mean difference in pain scores 12 h post-operation across studies (bubble size reflects sample size). Studies are plotted by year of publication, indicating trends in analgesic efficacy.

**Figure S2:**
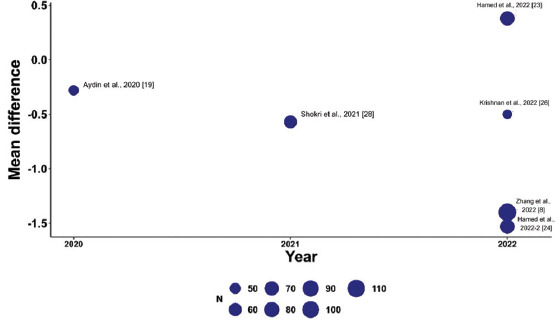
Meta-regression showing the mean difference in pain scores 24 h post-operation across studies (bubble size reflects sample size). Studies are plotted by year of publication, highlighting trends in analgesic efficacy over time.

**Figure S3:**
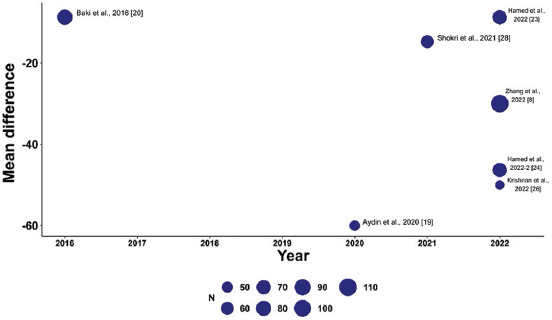
Meta-regression showing the mean difference in opioid consumption across studies (bubble size reflects sample size). Studies are plotted by year of publication, highlighting trends in opioid use reduction over time.

To explore variations in the observed effect size over the publication year, a scatterplot was created to display the effect size by time for each primary outcome. A substantial variability in sample sizes across studies was noted, as indicated by the size of the points on the plot, which are proportional to each study’s sample size. Figure S1 demonstrates that the MD in post-operative pain scores at 12 h decreased with each subsequent publication year, while Figures S2 and S3 show no clear association between effect size and study year. Meta-regression revealed no significant changes in primary outcomes when evaluated by procedure type or timing of the block. In addition, the funnel plots did not indicate asymmetry, suggesting an absence of a substantial small study effect (Figure S5).

**Figure S4:**
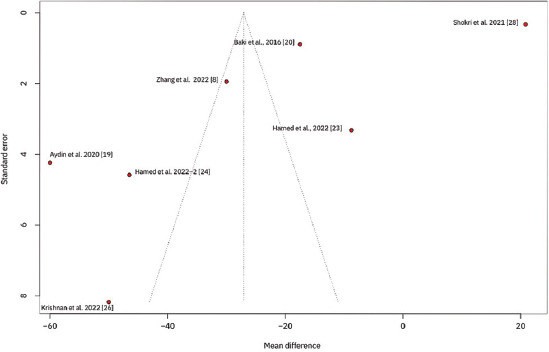
Funnel plot displaying the standard error against the mean difference for primary outcomes across studies. Each point represents a study, and the plot assesses potential publication bias by showing the distribution of effect sizes around the mean.

**Figure S5:**
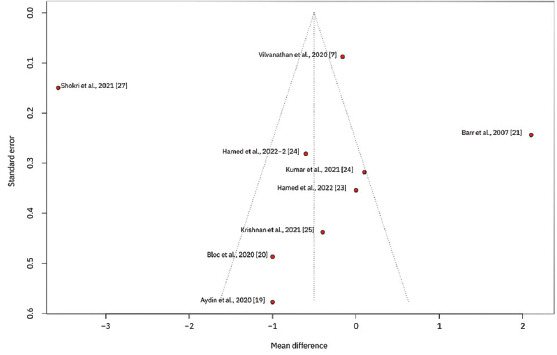
Funnel plot illustrating the standard error versus the mean difference in time to extubation across studies. Each point represents a study, with the plot assessing potential publication bias by showing the distribution of effect sizes around the average.

**Figure S6:**
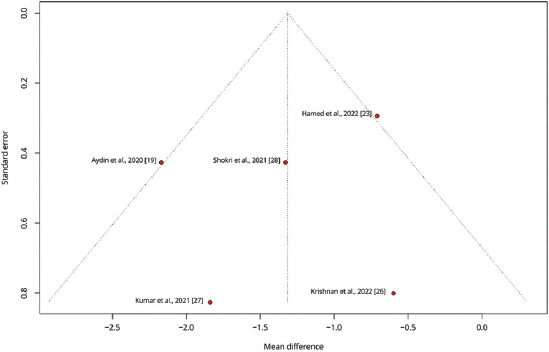
Funnel plot displaying the standard error against the mean difference for pain scores at 12 h post-operation across studies. Each point represents a study, with the plot assessing potential publication bias by showing the distribution of effect sizes around the mean.

**Figure S7:**
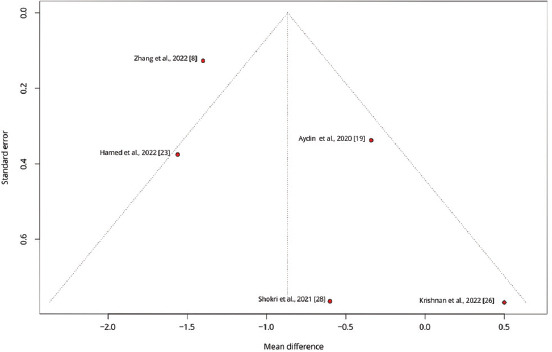
Funnel plot illustrating the standard error against the mean difference for pain scores at 24 h post-operation across studies. Each point represents a study, providing an assessment of potential publication bias by showing the distribution of effect sizes around the mean.

**Figure S8:**
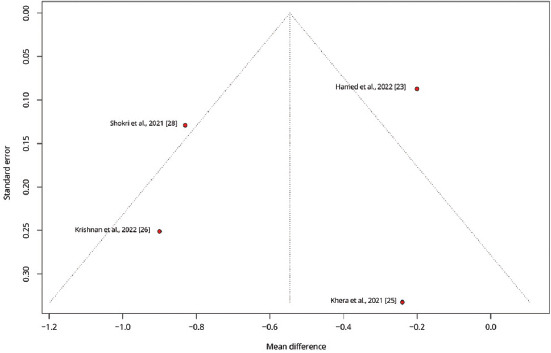
Funnel plot displaying the standard error against the mean difference for intensive care unit length of stay in days across studies. Each point represents a study, providing an assessment of potential publication bias by illustrating the distribution of effect sizes around the mean.

To assess the stability of the final pooled results, the authors employed a leave-one-out strategy. All outcome variables remained stable (Figures S4-S8).

### 3.3. Outcomes

#### 3.3.1. Primary outcomes

*3.3.1.1. Post-operative pain scores at 12 and 24 h*

**Figure 3:**
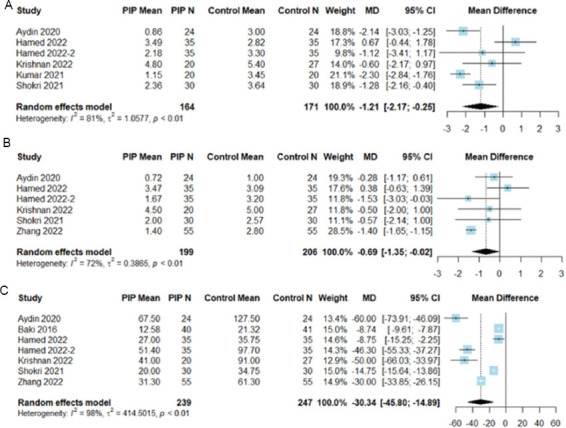
Forest plots for the primary outcomes of the included studies. (A) Post-operative pain scores at 12 h, measured on the visual analog scale ranging from 0 (no pain) to 10 (maximum pain). (B): Post-operative pain score at 24 h. (C): Morphine equivalent consumption on the 1st day.

Abbreviations: CI: Confidence interval; MD: Mean difference; N: Number of participants; PIP: Parasternal intercostal plane.

In six studies, patients reported post-operative pain scores at 12 h (Figure 3A) and 24 h (Figure 3B). Compared to the control group, the PIP block group demonstrated lower pain scores at 12 h post-surgery (MD: −1.21 points, 95% CI: −2.17, −0.25, *p* = 0.013, I^2^ = 81%) and at 24 h post-surgery (MD: −0.69 points, 95% CI: −1.35, −0.02, *p* = 0.042, I^2^ = 72%).

*3.3.1.2. Morphine equivalents*

Seven studies reported MME usage 24 h postoperatively (Figure 3C). Patients in the PIP block group consumed significantly less MME compared to the control group (MD: −30.34 MME, 95% CI: −45.80, −14.89, *p* < 0.001, I^2^ = 98%).

#### 3.3.2. Secondary outcomes

*3.3.2.1. Time to extubation*

**Figure 4:**
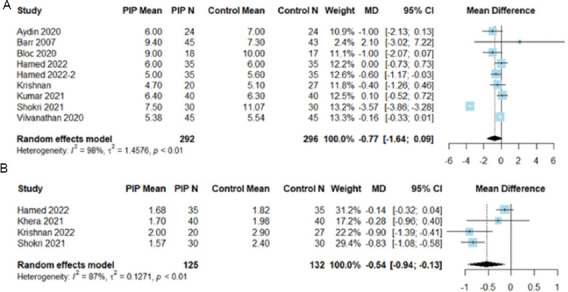
Forest plots for the secondary outcomes of the included studies. (A): Time to extubation, measured in hours. (B): Length of stay in ICU, measured in days.

Abbreviations: CI: Confidence interval; MD: Mean difference; N: Number of participants; PIP: Parasternal intercostal plane.

Nine studies evaluated time to extubation in hours. The PIP block group did not demonstrate a reduced time to extubation (MD: −0.77 h, 95% CI: −1.64, 0.09, *p* = 0.080, I^2^ = 98%) compared to the non-PIP group (Figure 4A).

*3.3.2.2. ICU length of stay*

Four studies reported ICU length of stay measured in days. The PIP block group exhibited a reduced length of stay in the ICU compared to the control group (MD: −0.54 days, 95% CI: −0.94, −0.13, *p* = 0.009, I^2^ = 87%) (Figure 4B).

## 4. Discussion

Our analysis revealed that PIP blocks resulted in a statistically significant reduction in post-operative pain scores and morphine equivalent consumption within the first 24 h after surgery. Prior studies have demonstrated that a single-shot PIP block effectively provided analgesia during the first 24 h in patients who underwent sternotomy,^18^,^27^ supporting its integration into multimodal anesthesia protocols.

The analgesic effect of PIP blocks operates by targeting the anterior cutaneous branches of T2 – T6. A recent cadaveric study by Lapisatepun *et al*.^10^ indicated that triple injections at the second, fourth, and fifth intercostal spaces for the superficial PIP block, along with double injections at the third and fifth intercostal spaces for the deep PIP block, resulted in consistent dye spread across the second to sixth intercostal spaces. This observation suggests effective coverage of the relevant nerves. However, it is important to recognize that pain following open cardiac surgeries is not confined to the sternotomy site; it may also arise from chest tube and vascular harvest sites, which could impact the primary outcomes reported in these studies. Our findings indicated that PIP block approaches can provide satisfactory analgesia despite the limitations of the anterior thoracic wall coverage. Our results build upon the recent network meta-analysis by Li *et al*., which similarly demonstrated the analgesic efficacy of this regional anesthesia technique for sternotomy.^28^

The analgesic effect of PIP blocks is particularly relevant given that acute pain following cardiac surgery typically peaks on the first post-operative day and gradually diminishes by the 3^rd^ day.^4^ By providing analgesia during the period of the most intense pain, PIP blocks can prevent a cascade of escalating analgesic requirements and reduce the risk of subacute pain conditions. Effective pain control is crucial to minimize the need for mechanical ventilation in open cardiac patients, which can subsequently decrease ICU length of stay. However, comparing ICU and ventilation outcomes is challenging due to variability in institutional protocols and guidelines across studies. For instance, Krishnan *et al*. reported a favorable ICU length of stay for the intervention group in our analysis, although this outcome did not reach statistical significance when compared to other groups within the same study. On the other hand, Shokri *et al*. linked ICU discharge with enhanced mobility and rehabilitation, which the regional anesthesia technique may have influenced. Nevertheless, we hypothesized that the analgesic effect of PIP blocks contributes to these short-term outcomes.

The reduced MME consumption observed in the intervention group highlights the advantages of employing regional anesthesia techniques across various procedures.^29^ This evidence also suggests that utilizing these techniques may decrease the incidence of chronic pain.^30^ The benefits of regional anesthesia can be attributed to the mechanisms of local anesthetics and their dispersion along fascial planes. However, ongoing debates revolve around the variability of these effects across different populations.^31^-^33^ Importantly, lower opioid doses have been found to be associated with better outcomes, including ameliorated post-operative nausea and vomiting and a lower incidence of cognitive dysfunction in various surgical procedures.^34^,^35^ These diverse benefits, ranging from improved pain scores to shorter discharge times, can enhance clinical practice, reduce healthcare costs, and mitigate the risk of pain and opioid-related side effects and adverse events.

Notably, complications were reportedly associated with PIP blocks when used as a part of a multimodal analgesic regimen. This suggests that techniques with lower safety profiles, such as the paravertebral block,^36^ may become less favored in anesthesia planning for anterior wall blocks, as considered in this study.

This systematic review and meta-analysis provided updated insights into the analgesic potential of the PIP block. Another recent meta-analysis by Li *et al*.^37^ explored similar techniques, such as the parasternal block, pectointercostal fascial plane block, and transversus thoracic muscle plane block; however, it did not include several blocks that fall under the definition of PIP according to the current nomenclature,^9^ such as parasternal intercostal block, superficial PIP block, modified parasternal intercostal block, and continuous pectointercostal fascial plane block. Our study, which included a more comprehensive set of RCTs, offered results that were both more precise and reliable compared to those of Li *et al.*,^37^ which likely lacked the full scope of our research focus. This discrepancy might explain why their study found no difference in the numeric rating scale at 24 h, while ours did.

Despite its strengths, this meta-analysis has limitations. The high level of heterogeneity in our data (I^2^ ranging from 72% to 99%) must be emphasized. This variability might stem from differences in block techniques (deep vs. superficial PIP), anesthetic volume and concentration, choice of anesthetic agent, timing of block administration, and post-operative analgesia regimens. Variability in proceduralist experience across studies might also contribute to elevated heterogeneity. In addition, the diverse outcomes in the included studies added to the study heterogeneity.38 Although our analysis was based on 12 RCTs, we presented reliable results with acceptable statistical rigor. Our study represents the largest sample size to date to compare PIP blocks across high-quality RCTs, favoring regional anesthesia over general anesthesia alone, despite the modest number of studies. While we focused on the analgesic effects of PIP blocks, we acknowledge that perioperative factors, such as comorbidities, surgical characteristics, and anesthetic methods likely influence extubation time and ICU length of stay.^39^ These variables were not considered in the included studies, potentially limiting the impact of PIP block on time to extubation.

To address potential bias, we utilized the ROB 2 tool to assess publication bias. Inconsistent blinding was noted, as some studies did not adequately blind outcome assessors and data analysts, which could introduce bias. In addition, the nature of ultrasound-guided procedures posed challenges for effective blinding. Due to the relatively small data set, we did not incorporate Egger’s test or other comparable measures to assess this bias.^40^

While we postulated that mechanistically all PIP target the T2 – T6 anterior cutaneous branches, it is plausible that different PIP block techniques may have unique mechanisms of action, warranting separate meta-analyses for each technique. The timing of block placement is another critical factor that may influence outcomes. Although our meta-regression did not reveal a significant impact of timing on primary outcomes, this remains an important area for future research.

## 5. Conclusion

Our study demonstrated that PIP blocks, whether administered as a deep or superficial technique, provided significant analgesic benefits and shortened ICU length of stay in cardiac surgeries involving sternotomy. However, given the considerable heterogeneity across the studies, these findings should be interpreted with caution. Further research utilizing more standardized protocols is essential to enhance our understanding of both the short- and long-term outcomes associated with PIP blocks.

## Data Availability

Data can be made available upon request to the authors and evaluated on a case-by-case basis.
